# Endothelin signalling in iridophore development and stripe pattern formation of zebrafish

**DOI:** 10.1242/bio.20148441

**Published:** 2014-05-23

**Authors:** Jana Krauss, Hans Georg Frohnhöfer, Brigitte Walderich, Hans-Martin Maischein, Christian Weiler, Uwe Irion, Christiane Nüsslein-Volhard

**Affiliations:** Max-Planck-Institut für Entwicklungsbiologie, 72076 Tübingen, Germany

**Keywords:** *karneol*, Iridophores, Endothelin-converting enzyme, Endothelin signalling, Pigment pattern formation

## Abstract

Colour patterns of adult fish are composed of several different types of pigment cells distributing in the skin during juvenile development. The zebrafish, *Danio rerio*, displays a striking pattern of dark stripes of melanophores interspersed with light stripes of xanthophores. A third cell type, silvery iridophores, contributes to both stripes and plays a crucial role in adult pigment pattern formation. Several mutants deficient in iridophore development display similar adult phenotypes with reduced numbers of melanophores and defects in stripe formation. This indicates a supporting role of iridophores for melanophore development and maintenance. One of these mutants, *rose* (*rse*), encodes the Endothelin receptor b1a. Here we describe a new mutant in zebrafish, *karneol* (*kar*), which has a phenotype similar to weak alleles of *rse* with a reduction in iridophore numbers and defects of adult pigment patterning. We show that, unlike *rse*, *kar* is not required in iridophores. The gene defective in the *kar* mutant codes for an endothelin-converting enzyme, Ece2, which activates endothelin ligands by proteolytic cleavage. By morpholino-mediated knockdown, we identify Endothelin 3b (Edn3b) as the ligand for endothelin receptor signalling in larval iridophores. Thus, Endothelin signalling is involved in iridophore development, proliferation and stripe morphogenesis in larvae as well as adult zebrafish. In mammals the pathway is required for melanocyte development; therefore, our results indicate a previously unrecognized close evolutionary relationship between iridophores in zebrafish and melanocytes in mammals.

## INTRODUCTION

Adult zebrafish display a characteristic body and fin pigmentation pattern of alternating dark stripes and light interstripes. Three pigment cell types are known to build this pattern: black melanophores, yellow xanthophores and silvery iridophores. Melanophores covered by loose “blue” iridophores dominate the dark stripes, whereas dense iridophores covered by xanthophores generate the light stripes (Frohnhöfer et al., 2013; Hirata et al., 2003; Hirata et al., 2005). These pigment cells are derived from the neural crest, a transient embryonic structure in vertebrates which also contributes to many other tissues, e.g. bone, cartilage and the enteric nervous system (Le Douarin and Dupin, 2003). In zebrafish the larval pigmentation pattern is composed of iridophores and melanophores arranged into a dorsal, a ventral and a yolk-sac stripe, whereas melanophores align along the horizontal myoseptum into lateral stripes; xanthophores cover the entire larval body (Eisen and Weston, 1993; Kelsh, 2004; Raible and Eisen, 1994). The adult striped pattern develops during metamorphosis mainly by newly differentiating pigment cells in the dermis (Johnson et al., 1995; Kirschbaum, 1975; Parichy, 2003; Parichy et al., 2009). Iridophores reach the dermis at the horizontal myoseptum, proliferate and spread to create a series of dense ridges of interstripes that serve melanophores to accumulate into stripes (Frohnhöfer et al., 2013; Singh et al., 2014). Xanthophores closely follow iridophores in stripe development. Melanophore progenitors proliferate while migrating along spinal nerves into the skin where they melanise to form the dark stripes. Both iridophores and melanophores are derived from segmentally arranged postembryonic stem cells located close to dorsal root ganglia while the origin of metamorphic xanthophores is not known (Budi et al., 2011; Dooley et al., 2013; Hultman and Johnson, 2010; Singh et al., 2014).

Recently, a crucial role of iridophores in stripe formation in the skin of the body was demonstrated. Mutants deficient in iridophores show a strong reduction of melanophore numbers and defects in stripe formation (Frohnhöfer et al., 2013; Krauss et al., 2013; Patterson and Parichy, 2013). Four genes were so far found to be required for iridophore development, *shady* (*shd*) encoding Leukocyte tyrosine kinase (Ltk), *rose* (*rse*) encoding Endothelin-receptor b1a (Ednrb1a), *transparent* (*tra*) encoding the mitochondrial protein Mpv17 and *bonaparte* (*bnp*) encoding the transcription factor basonuclin-2 (bnc-2) (Krauss et al., 2013; Lang et al., 2009; Lopes et al., 2008; Parichy et al., 2000). In strong alleles of *shd* or *rse* only the first stripes, 1D and 1V, develop, albeit broken into spots; in weak alleles, some residual interstripe formation is observed, and the striped organization of the melanophores is better preserved. Chimeric animals obtained by blastula transplantations revealed that in the case of *shd*, *rse* and *tra* the genes are autonomously required in iridophores, while mutant melanophores and xanthophores are not affected and can form normal stripes when confronted with wild-type iridophores (Frohnhöfer et al., 2013; Krauss et al., 2013). This indicates that in all three cases the loss of iridophores causes the observed complex phenotypes, and that differentiated iridophores sustain melanophore development and stripe formation. In contrast to this, *bnp* seems to be required in a non-chromatophore cell type (Lang et al., 2009; Patterson and Parichy, 2013).

Both *shd* and *rse* encode plasma membrane receptor proteins. Ltk belongs to the class of receptor tyrosine kinases; it is expressed broadly in pre-migratory neural crest cells with gradual restriction to developing iridophores during later embryonic stages. Mutations in *shd* lead to a lack of iridophores throughout all developmental stages, which led to the assumption that Ltk function is required for the specification of iridophores (Lopes et al., 2008). Ednrb1a belongs to a family of G-protein coupled receptors. Mutations in *rse*, although the gene is expressed in pigment cells during early zebrafish development, do not show defects in embryonic iridophores (Parichy et al., 2000), possibly due to redundancy, as zebrafish contain a second paralog, *ednrb1b*. For both receptors, the activating ligands have not been identified yet in zebrafish. In mammals, where Endothelin signalling promotes the development of melanocytes, mutations in Ednrb or its ligand Endothelin 3 (Edn3) lead to a reduction of melanocytes, as well as aganglionosis caused by a strong reduction in sensory gut neurons (Baynash et al., 1994; Gariepy et al., 1996; Hosoda et al., 1994; Kunieda et al., 1996; Metallinos et al., 1998; Santschi et al., 1998). Similar reductions in the number of melanocytes were observed in mice carrying a knock-out allele of the endothelin-converting enzyme 1 (*Ece1*). This enzyme cleaves the inactive Endothelin precursor proteins to produce the biologically active 21-aa peptide ligands (Yanagisawa et al., 1998). In humans, mutations in *EDN3* and *EDNRB* cause Waardenburg-Shah syndrome (Waardenburg syndrome type IV) hallmarked by defects in pigmentation and neonatal bowel obstructions (Amiel et al., 1996; Edery et al., 1996; Hofstra et al., 1996).

Here we investigate the function of the endothelin pathway in pigment pattern formation in zebrafish. We describe *karneol* (*kar*), a new iridophore-deficient mutant that shows an adult-specific phenotype similar to weak *rse* alleles. Chimeric animals reveal that in contrast to *rse*, *shd* and *tra*, *kar* is not required in iridophores or in any other chromatophore type. We identified a mutation in the endothelin-converting enzyme 2 (*ece2*) gene resulting in a premature stop and the loss of the C-terminal peptidase domain containing the catalytic centre of the enzyme. As neither *rse* nor *kar* mutants develop pigmentation defects in larvae, we investigated the role of endothelins in larval pigmentation. By expression analysis and morpholino knockdown we identify Edn3b as potential ligand of *rse* acting in iridophore development in zebrafish. Thus Edn3 signalling, which in mammals is involved in melanocyte development, affects specifically iridophores in zebrafish.

## MATERIALS AND METHODS

### Fish husbandry

Zebrafish were maintained as described earlier (Brand et al., 2002); we used the following genotypes: *karneol^tNO046^*, *transparent^b6^*, *rose^tLF802^*, *rose^tAN17X^*, *nacre^w2^*, *pfeffer^tm236b^*, Tuebingen, *albino^b4^*, *Tg(β-actin:GFP)* and WIK. Embryos and larvae were staged as described previously (Kimmel et al., 1995). Staging of juveniles was done according to Parichy et al. (Parichy et al., 2009). The experiments with zebrafish conform to the regulatory standards relevant for Baden-Württemberg, Germany.

### Transplantations

Chimeric animals were generated as described previously (Kane and Kishimoto, 2002).

### Genetic mapping

Genetic linkage was determined as described previously (Geisler et al., 2007). For validation of the candidate gene, *ece2*, genomic DNA or total RNA was prepared from fin clips using TRIzol Reagent (Invitrogen) according to manufacturer's protocol. Reverse transcription was performed using total RNA and the Omniscript RT kit (Qiagen). The following primers were used:

T1315: 5′-GAGAGCTGATCTCTATCTATCTCC-3′

T1316: 5′-GCTTGAGAAAGAGCCACAAC-3′

T1317: 5′-AGAGAGGAAGACACAGTCG-3′

T1318: 5′-ATGACCACTCCAATCCCAC-3′

T1319: 5′-CATCAACAAGACCGACCAC-3′

T1320: 5′-CTCCTTTCTGCACCAGATTC-3′

T1321: 5′-CGACAGTGAACGCTTACTAC-3′

T1322: 5′-TCAAATCCATGAGTGGTTGG-3′

T1357: 5′-GATCAATGAAATCCGCACG-3′

T1358: 5′-CATCATACACATCATCCAGCTC-3′

### Phylogenetic analysis

The alignment of amino acid sequences was generated using ClustalW. Sequences with the following accession numbers were used: Ece1 human: NP_001388.1; Ece1 mouse: NP_955011.1; Ece1 chick: NP_990048.1; Ece1 zebrafish: NP_001071260.1; Ecel1 human: NP_004817.2; Ecel1 mouse: NP_067281.2; Ecel1 chick: XP_422744.3; Ecel1 zebrafish: ENSDARG00000060549; Ece2 human: NP_055508.3; Ece2 mouse: NP_808810.1; Ece2 chick: XP_003641814.1; Ece2 zebrafish: KJ622365. The phylogenetic tree was calculated with PHYLIP-NEIGHBOR from the MPI Bioinformatics Toolkit (http://toolkit.tuebingen.mpg.de/phylip) using 100 bootstrap repetitions.

### RNA *in situ* hybridization

Templates for RNA probes were amplified by RT-PCR using an antisense DNA oligo containing a T7 promoter sequence on its 5′ end. The following oligos were used:

edn3b: 5′-CATCATCTGGATCAACAC-3′ and 5′-TGGATCCTAATACGACTCACTATAGGGCAAGGTGAACGTCCTCTC-3′

edn3a: 5′-CGTCCTGAAGCGCTCGTG-3′ and 5′-TGGATCCTAATACGACTCACTATAGGGATGTGCAGTCCTGGTC-3′

pnp4a: 5′-GCACTGTGCTGGCTTCCAC-3′ and 5′-TGGATCCTAATACGACTCACTATAGGGGCTGTTATGGCTGATCCTC-3′

DIG-labelled probes were generated by *in vitro* transcription with T7 RNA polymerase using the DIG-RNA labelling-Mix (Roche). RNA *in situ* hybridization was carried out according to standard procedures.

### Image acquisition

Adult fish were briefly anaesthetized with 0.004% MS-3222 (Sigma) or fixed over night at 4°C in 4% PFA/1% glutaraldehyde and imaged with Canon D5MarkII/Macro 100. The angle of illumination had to be adjusted individually to allow optimal visualization of iridophore pigmentation. Images of larvae were taken on a Discovery stereo microscope (Zeiss). Photographs were processed in Adobe Photoshop.

### Morpholino injections

The following antisense morpholinos for *edn3b* were obtained from Gene Tools:

edn3b AUG-MO: 5′-GTGCATCAGGAATCAGTTTAGCCAT-3′

edn3b splice-MO: 5′-TCAGTCAGCAAAAGCACTTACCCAC-3′

edn3b splice mismatch-MO: 5′-TCACTCACCAAAACCAGTTAGCCAC-3′

Morpholinos were injected into Tuebingen and *alb^b4^* one-cell stage embryos. Embryos were either fixed at 48 hpf and subjected to RNA *in situ* hybridization or analyzed at 5 dpf for pigment cell defects. Pools of 10 injected or control embryos were used for RT-PCR to test the efficiency of the splice morpholino using the following primer pairs:

edn3b forward: 5′-GATGAGGATGCTCAGAAC-3′ and

edn3b reverse: 5′-GTGTTGATCCAGATGATG-3′

eef1a1 forward 5′-GAGGAAATCACCAAGGAAGTC-3′ and

eef1a1 reverse 5′-AGGTCACAACCATACCAGGC-3′

## RESULTS AND DISCUSSION

### Adult *kar* mutants are deficient in iridophores and melanophores

**Fig. 1.**
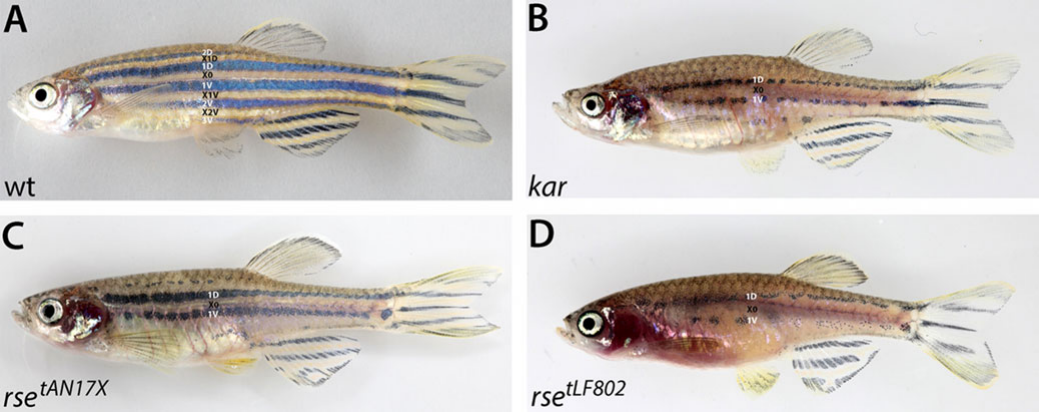
*kar* mutants display reductions in iridophores and melanophores similar to weak *rse* mutants. Wild-type (A), *kar* (B), weak *rse* (*rse ^tAN17X^*) (C) and strong *rse* mutant (*rse^tLF802^*) (D) adult fish. The stripes (2D, 1D, 1V, 2V, 3V) and interstripes (X1D, X0, X1V, X2V) are indicated. Weak *rse* (C) and *kar* (B) display similar reductions in iridophores and melanophores as well as defects in the stripe pattern.

In an ENU mutagenesis experiment for adult pigmentation phenotypes we identified a mutant with a strong reduction of iridophores and melanophores (Fig. 1A,B), which we named *karneol* (*kar*) after the semi-precious stone. The *kar* mutant phenotype is similar to that of weak *rse* alleles, e.g. *rse^tAN17X^* (Fig. 1C), whereas strong *rse* alleles, e.g. *rse^tLF802^*, lead to an even further reduction of iridophores and melanophores (Fig. 1D). Both, *kar* and weak *rse* mutants develop two dark stripes, 1D and 1V, containing fewer melanophores than wild type and only remnants of 2D and 2V. Due to these similarities we performed complementation analysis between *rse* and *kar*; double heterozygotes show no mutant phenotype and we conclude that we identified a new locus required for iridophore development in adult zebrafish.

**Fig. 2.**
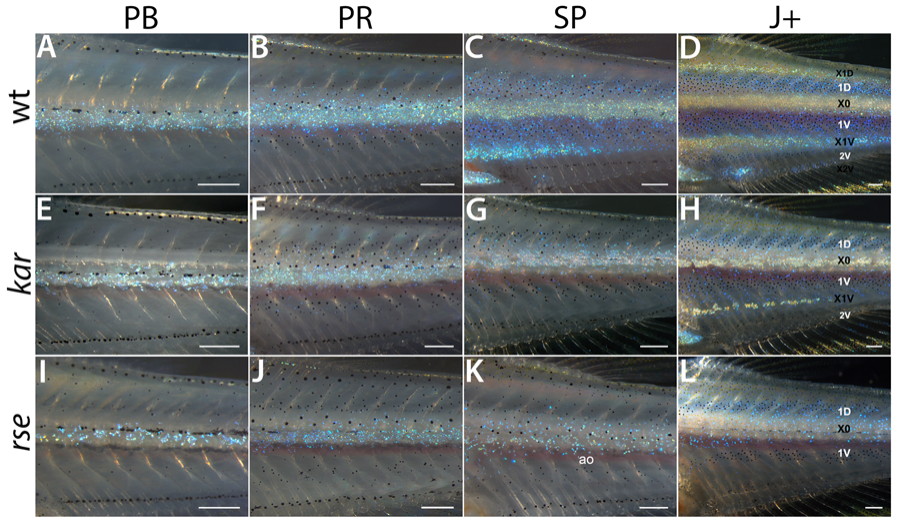
The *kar* phenotype arises during metamorphosis. Developmental series of body pigment pattern metamorphosis of wild-type (A–D), *kar* (E–H) and *rse^tLF802^* mutant fish (I–L). Posterior trunk regions at the level of dorsal and anal fins are shown. The series of wild type and strong *rse* were published by Frohnhöfer et al. (Frohnhöfer et al., 2013). At stages PB and PR, *kar* mutants show only slight reductions in iridophore numbers (compare panels A,B to panels E,F), they still form a continuous sheet just ventral to the horizontal myoseptum. From PR to SP however, this reduction of iridophores becomes more apparent. They do not extend properly into dorsal and ventral interstripe regions (compare panel C and panel G), occasionally, remnants or patches develop (interstripe X1V in panel H). From stage SP onwards the number of melanophores is reduced compared to wild type (compare panels C,D to panels G,H). The early phase of pigment pattern development of strong *rse* mutants is similar to *kar*, with slightly stronger reductions in iridophores (I,J). In contrast to *kar*, however, iridophores do not form dense ridges in interstripe regions and rather are dispersed (K,L). ao: aorta. Scale bars: 250 µm.

Metamorphosis of the body pigmentation pattern in *kar* mutants starts similar to wild type with the appearance and proliferation of iridophores in X0. At stage PB (Fig. 2A,E) there is a slight reduction in their number, but less pronounced than in strong *rse* mutants (Fig. 2I). This reduction becomes more apparent later in metamorphosis (Fig. 2A–H). In addition, from stage SP onwards the number of blue iridophores that spread dorsally and ventrally during juvenile stages is reduced in *kar* mutants (Fig. 2C,G). Unlike in strong *rse* mutants iridophores in *kar* mutants form thin ridges in the middle of the interstripe regions X0 and X1V (Fig. 2H,L). These ridges typically persist into adulthood in X0, while those in X1V dissolve during later stages. Similar to strong *rse* mutants, *kar* mutants show a reduction in the number of melanophores from stage SP onwards (Fig. 2C,G,K). In conclusion, both *kar* and *rse* are required for iridophore proliferation and aggregation during stripe formation, which argues for a role of *kar* in the Edn signalling pathway.

### *kar* encodes Endothelin-converting enzyme 2

**Fig. 3.**
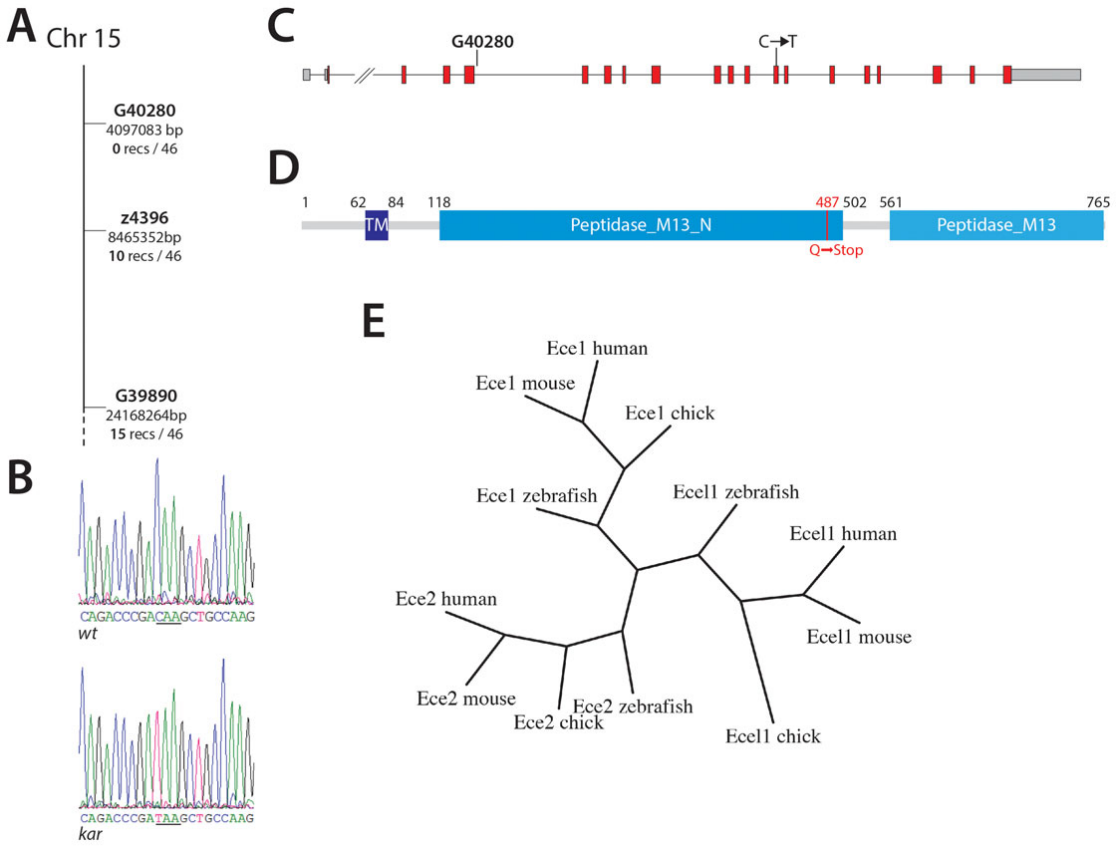
*kar* encodes endothelin-converting enzyme 2. *kar* maps to a region at the beginning of chromosome 15 (A). One marker, G40280, is 100% linked to the mutation and lies in the gene coding for endothelin-converting enzyme 2 (A,C). Chromatograms of *ece2* sequences from wild-type and *kar* mutant fish (B) showing the mutation (the premature Stop codon is underlined). Ece2 is a type II transmembrane protein of 765 aa (D), the C-terminal peptidase domain is lost in the mutants, TM: transmembrane domain. Phylogenetic analysis of the amino acid sequences of Eces from different vertebrate species shows three branches with Ece1, Ecel1 and Ece2 (E).

To identify the molecular lesion causing the *kar* mutant phenotype, we mapped the mutation using microsatellite markers. Three markers on chromosome 15 showed strong linkage with the mutant phenotype. One marker, G40280, was fully linked with no recombinant amongst the 46 mapping fish; the other two markers, z4396 and G39890, were less closely linked with 10 and 15 recombinants, respectively (Fig. 3A). This places the mutation on the beginning of chromosome 15 in an interval of less than 8.5 Mbp. The fully linked marker G40280 lies in an intron of the gene encoding Endothelin-converting enzyme 2 (Ece 2), which is a strong candidate for *kar*. We sequenced cDNA of this transcript (GenBank accession: KJ622365), which corresponds to the Ensembl gene RNASEQG00000017608 without the long 3′ UTR. At bp 1456 of the CDS (genomic position Zv9:15:4049885) we identified a C to T substitution, which leads to a premature stop codon resulting in a truncation of the predicted protein at amino acid 487 with the complete loss of the C-terminal thermolysin-like peptidase domain, which contains the zinc-coordinating residues and the catalytic centre of the enzyme (Fig. 3B–D). A phylogenetic analysis of Endothelin-converting enzymes from several vertebrates (human, mouse, chick and zebrafish) confirmed that the gene encodes indeed the zebrafish homologue of Ece2 (Fig. 3E). To further demonstrate that the identified base substitution is indeed the mutation causing the *kar* phenotype, and not simply a polymorphism in the zebrafish genome without further consequence, we sequenced the site in 49 wild-type fish from different backgrounds. All of them showed a C at the relevant position. In addition we also sequenced the progeny of heterozygous *kar* mutant incrosses. All fish with the *kar* phenotype were homozygous for the identified substitution (*n* = 96). Therefore we consider *kar* to be a loss-of-function allele of *ece2*.

### *kar* is not required in iridophores nor other chromatophores

**Fig. 4.**
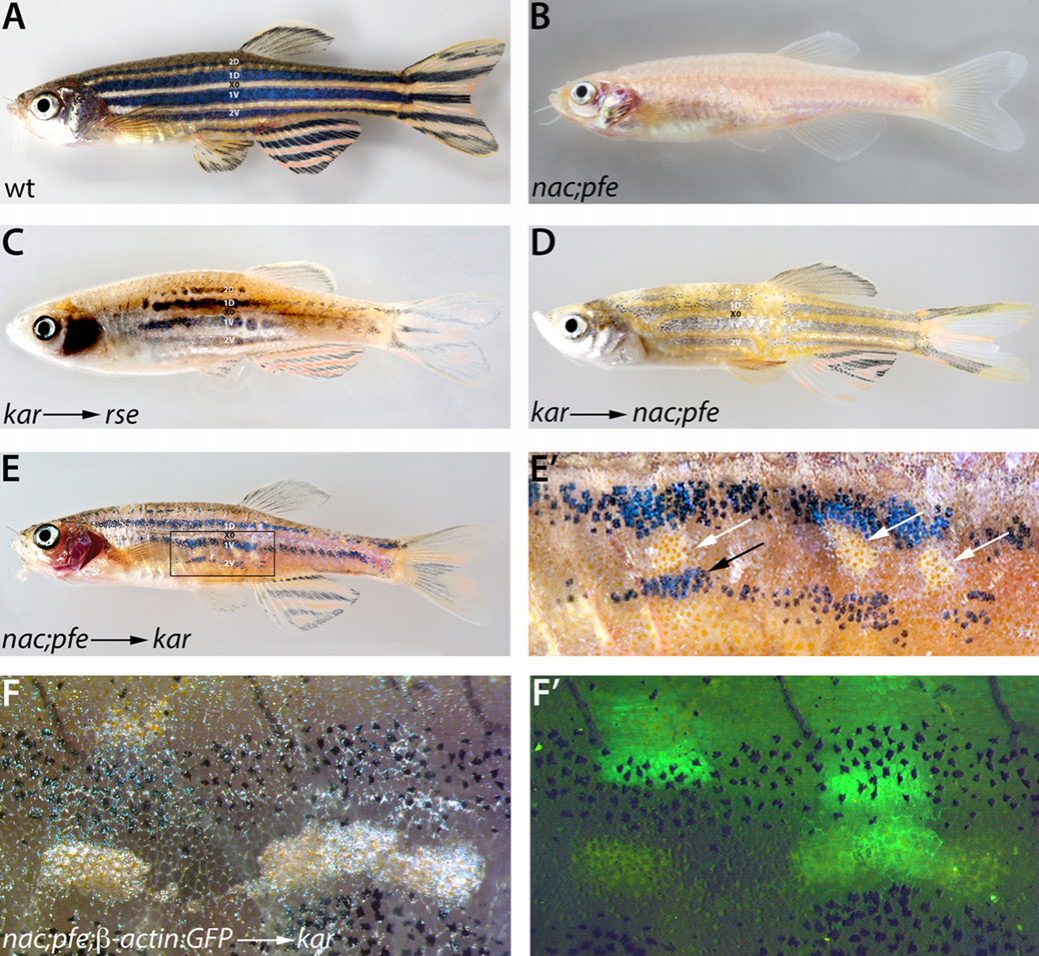
The *kar* gene product, Ece2, acts outside pigment cells to promote iridophore development. Wild-type (A) and *nac;pfe* mutant (B) adult fish for comparison. Transplantations of *kar* mutant cells into strong *rse* (C) or *nac;pfe* (D) mutant recipients result in fish with recovered stripe patterns. In chimeric fish generated by transplantation of *nac;pfe* donor cells into *kar* mutant recipient embryos (E,E′) occasionally, very small patches of dense iridophores develop (magnification in E′, white arrows). In the vicinity of these patches melanophores increase in number (black arrow). Labelling of the donors with Tg(β-actin:GFP) (F,F′) shows transplanted donor cells of various cell types next to the patches of dense iridophores.

The *rse* and *shd* gene products are required within iridophores, but not in melanophores nor xanthophores (Frohnhöfer et al., 2013). The similarities of *kar* with weak *rse* alleles prompted us to test if *kar* is also required in iridophores, or in any other chromatophore type. To this end we transplanted cells between embryos of different genotypes during the blastula stage. Transplantations of *kar* cells into *rse* hosts resulted in chimaeras with large patches of wild-type pattern and normal sized stripes (Fig. 4C). This indicates that *kar* mutants provide normal iridophores to *rse* mutant fish, and that *kar* is not required in iridophores.

Using *nac;pfe* mutant hosts (Fig. 4B) the resulting chimaeras, after transplantations of *kar* mutant cells, also developed normally patterned regions (Fig. 4D). Here the only host-derived pigment cells are iridophores, due to mutations in *nacre/mitfa*, required for melanophore development, and in *pfeffer/fms*, which acts in xanthophores. The rescue of the pigmentation pattern in this situation indicates that *kar* mutant melanophores and xanthophores are not affected and behave normally when confronted with wild-type host cells, as is the case for *rse* (Frohnhöfer et al., 2013). However, transplantations of cells from *nac;pfe* into *kar* mutant hosts led only to the development of very small patches of dense iridophores and some local improvement of the *kar* residual pigment pattern (Fig. 4E,E′), showing that iridophores can only poorly develop in the *kar* mutant environment. Transplantations of GFP-marked cells from *nac;pfe* mutants into *kar* hosts showed donor-derived GFP-positive cells in the vicinity of these small patches of dense iridophores without all iridophores being labelled.

Taken together, these results demonstrate that there is no cell autonomous requirement for *kar* activity in any of the chromatophores. Instead *kar* promotes iridophore development in a non-cell autonomous manner, which is in agreement with the molecular nature of Ece2 as the identified *kar* gene product. Enzymes of this class proteolytically cleave inactive Pro-endothelins to produce active Edns. These secreted peptide ligands then bind to receptors, Ednrs, located in the plasma membranes of target cells and activate down-stream signalling. Our data suggest a paracrine mode of endothelin signalling during pigment patterning. Iridophores express the receptor Ednrb1a (Lang et al., 2009), the *rse* gene product, which in *kar* mutant embryos cannot be fully activated due to the lack of processed ligand. The ligand and the processing enzyme, Ece2, are likely to be produced by non-pigment cells in the vicinity of the iridophores. However, we cannot rule out that in the wild-type situation iridophores also express *ece2* and contribute to the signalling in an autocrine manner.

There is considerable redundancy in the endothelin pathway in vertebrates. In zebrafish six genes encode Edn ligands, five Ednrs and three Eces (Braasch et al., 2009). The differences in the severity of the phenotypes in *rse* and *kar* mutants could be explained by this redundancy. One of the other two enzymes could also process ligand in the vicinity of iridophores leading to some residual signalling activity via the *rse* receptor in *kar* mutants. Redundancy and/or sub-functionalization could also explain the lack of defects during larval development in *rse* and *kar* mutants, where other receptors and enzymes might function.

### Edn3b is required for larval iridophore development

**Fig. 5.**
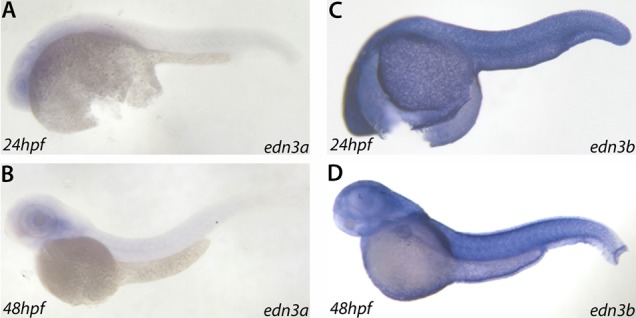
*edn3b* is expressed in the epidermis during early development. RNA *in situ* hybridizations for *edn3a* and *edn3b* at 24 hpf and 48 hpf in *albino* (*alb*) embryos. The expression of *edn3a* is not detectable above background (A,B). *edn3b* expression is detected in the epidermis during these stages (C,D).

**Fig. 6.**
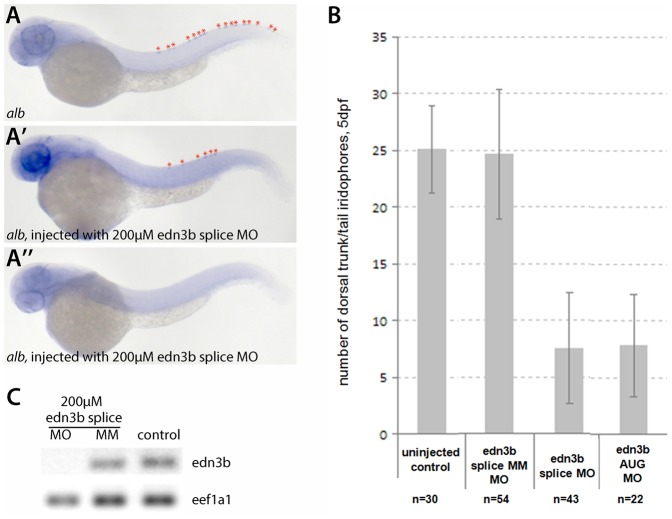
Morpholino-mediated knockdown of *edn3b* function in wild-type and *alb* embryos results in a strong reduction of iridophores. (A–A″) Morpholinos designed to interfere with splicing or translation of *edn3b* were injected into *alb* embryos, RNA *in situ* hybridization for *pnp4a* at 48 hpf shows a reduction of iridophore numbers in the dorsal stripes (marked with asterisks in panels A and A′). At 5 dpf morpholino injected larvae show strong reductions in iridophore numbers. Injection of a 5-bp-mismatch control morpholino had no effect (B). RT-PCR results to detect *edn3b* spliced message (C). Whereas the mismatch control morpholino showed no effect on the level of *edn3b* transcript, the PCR did not result in amplification of *edn3b* message after injection of the splice-interfering morpholino.

In mammals Ednrb and Edn3 are involved in the development of melanocytes (Baynash et al., 1994). We investigated the expression of the two zebrafish *edn3* paralogs during larval stages. The expression of *edn3a* at 24 hpf and 48 hpf is not detectable above background, whereas *edn3b* is strongly expressed in the epidermis (Fig. 5). To examine a potential role of *edn3b* in pigment cell development, we performed knockdown experiments by injection of morpholinos into one-cell stage embryos. Injection of morpholinos targeting either the translation start site or a splice site of *edn3b* pre-mRNA, led to a significant reduction of iridophores at 48 hpf, as measured by RNA in situ hybridization using *pnp4a* as an early and specific marker for iridophores (Lang et al., 2009) (Fig. 6A). We further analysed this phenotype by counting iridophores based on their reflective properties in the dorsal trunk of 5 dpf larvae (Fig. 6B). Both morpholinos resulted in similar reductions of iridophores at 5 dpf. The control 5 bp mismatch splice morpholino did not show such an effect. We did not detect defects in melanophore development in the larval pattern (data not shown). RT-PCR analysis of *edn3b* confirmed the activity of the splice morpholino (Fig. 6C). These results suggest that Edn3b is specifically required for iridophore but not for melanophore development during early larval stages. In contrast to this, edn3 signalling in mammals is specifically required for melanocyte migration and maintenance in dermis and epidermis and the establishment of the enteric nervous system. In zebrafish there is evidence that a fraction of the adult melanophores share a common precursor with iridophores (Singh et al., 2014). It is conceivable that this population is homologous to melanocytes in amniotes. However, the majority of melanophores in zebrafish are independent of iridophores and we suspect that other paralogs of genes coding for endothelin signalling components are involved in their development.
